# Bilateral inguinal vesicles clustered on an ecchymotic background

**DOI:** 10.1016/j.jdcr.2024.07.036

**Published:** 2024-08-24

**Authors:** Frank Z. Jing, Michael Camilleri, Julio C. Sartori-Valinotti

**Affiliations:** a Department of Dermatology, Mayo Clinic, Rochester, Minnesota; b Division of Dermatopathology, Department of Dermatology, Mayo Clinic, Rochester, Minnesota

**Keywords:** cancer, colorectal adenocarcinoma, cutaneous metastasis, immunohistochemistry, lymphangioma, lymphatic

## History

**Fig 1:**
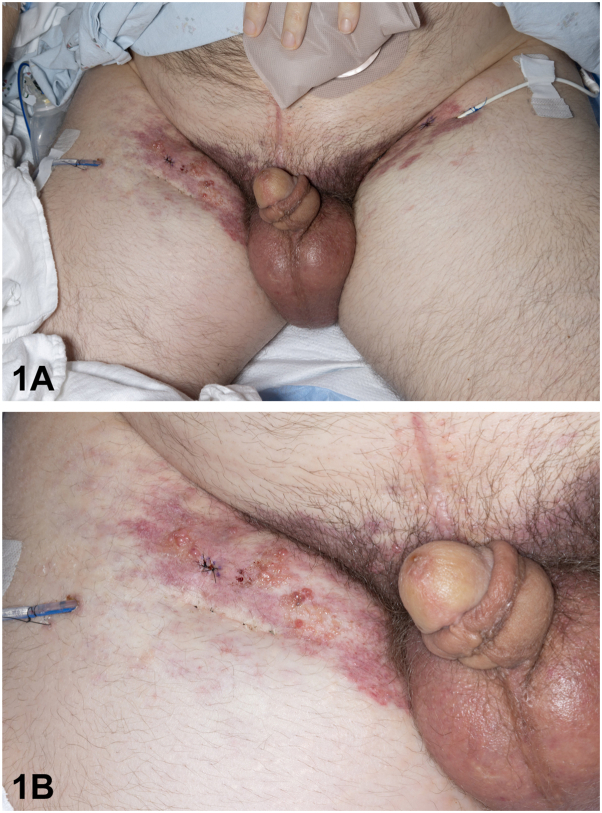

**Fig 2:**
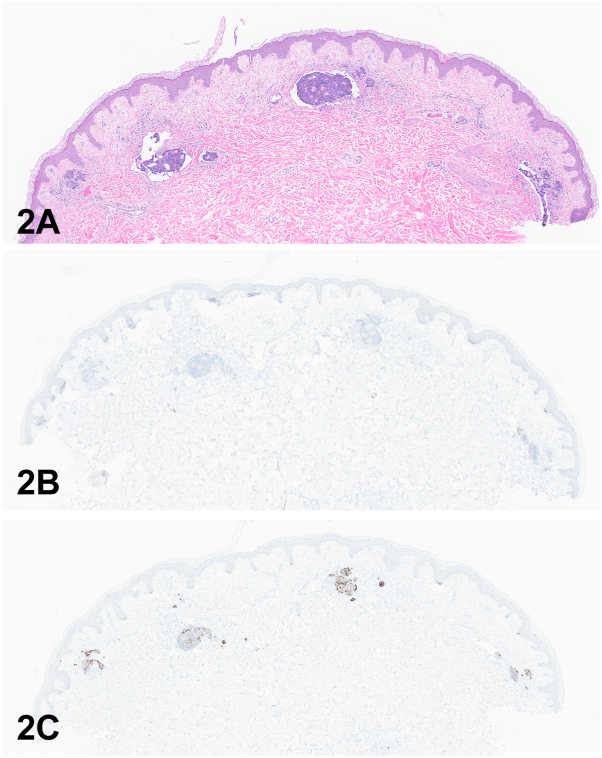

A male in his 40s with history of metastatic colorectal adenocarcinoma who underwent abdominoperineal resection, chemotherapy, and pelvic lymphadenectomy was admitted for anasarca and progressive bilateral lower extremity lymphedema following drain placement for inguinal seromas. On physical exam, bilateral inguinal ecchymotic indurated plaques with superimposed clusters of translucent tense vesicles were noted with extension to the anterior thighs (Fig 1, *A* and *B*). Punch biopsies performed from each inguinal region revealed lesions comprised of pleomorphic cells with prominent nucleoli and glandular differentiation (Fig 2, *A*). Immunohistochemical studies were negative for cytokeratin (CK) 7 and positive for CK 20 (Fig 2, *B* and *C*).

**Question 1: What is the diagnosis?**

A. Angiosarcoma
B. Changes secondary to pelvic lymphadenectomy
C. Lymphangioma
D. Herpes zoster
E. Recurrence and cutaneous metastasis of colorectal adenocarcinoma

**Answers:**

A. Angiosarcoma – Incorrect. While the history of radiation, lymphedema, and the presence of ecchymotic plaques would place angiosarcoma on a differential diagnosis, it would not explain the presence of grouped vesicles or histology findings.
B. Changes secondary to pelvic lymphadenectomy – Incorrect. The previous pelvic lymphadenectomy does explain the patient’s seromas and lymphedema but does not explain the ecchymosis and presence of tumor cells on pathology.^1^
C. Lymphangioma – Incorrect. While the grouped serosanguinous fluid-filled vesicles clinically and dilated lymphatic channels histologically have similarity with lymphangioma circumscriptum, the presence of tumor in lymphatic channels argues against this diagnosis.
D. Herpes Zoster – Incorrect. While the herpetiform appearance, dermatomal distribution, and history of varicella favor the diagnosis, the tense, long-lived, asymptomatic nature of the lesions and lack of viral cytopathic changes in histology argue against herpes zoster.
E. Recurrence and cutaneous metastasis of colorectal adenocarcinoma – Correct. The patient’s history of metastatic colorectal adenocarcinoma, presence of indurated ecchymotic plaques, progression of tense long-lived vesicles, and malignancy engorging the lymphatic channels confirm recurrence and cutaneous metastasis of his colorectal adenocarcinoma.

**Question 2: Which of the following statements is true?**

A. Approximately 10% of cutaneous metastasis cases have herpetiform, zosteriform, or lymphangioma-like patterns.
B. Approximately two-thirds of patients with cutaneous metastasis of internal malignancy survive past 6 months but die within 1 year.
C. Cutaneous metastasis is a common presenting sign of internal malignancy.
D. Over 5% of cutaneous metastasis cases arise 5 or more years after the primary cancer diagnosis.
E. The most common immunophenotype for colorectal adenocarcinoma is positive CK7 and CDX2 and negative CK20.

**Answers:**

A. Approximately 10% of cutaneous metastasis cases have herpetiform, zosteriform, or lymphangioma-like patterns – Incorrect. Herpetiform, zosteriform, and lymphangioma patterns are exceedingly rare presentations of cutaneous metastasis, which previously led clinicians to attempt treatment with antimicrobials, delaying definitive diagnosis. Cutaneous metastasis most commonly presents as nodules or masses in 80.5%.^2^
B. Approximately two-thirds of patients will survive past 6 months but die within 1 year – Incorrect. More than two-thirds of patients will die within the first 6 months.^3^
C. Cutaneous metastasis is a common presenting sign of internal malignancy – Incorrect. Cutaneous metastasis represents a rare phenomenon overall in patients with internal malignancy, with an incidence of approximately 2%.^2^
D. Over 5% of cutaneous metastasis cases arise 5 or more years after the primary cancer diagnosis – Correct. In approximately 7% of cases, the interval between primary diagnosis and cutaneous metastasis is greater than 5 years.^2^
E. The most common immunologic staining pattern for colorectal adenocarcinoma is positive CK7 and CDX2 and negative CK20 – Incorrect. The most common immunophenotype is positive CK20 and CDX2 and negative CK7. CDX2 is an expressed transcription factor in the nuclei of epithelial cells in the intestine, duodenum, and rectum.^4^

**Question 3: What is the most likely explanation for cutaneous dissemination of colorectal cancer in this patient?**

A. Direct extension from primary colorectal carcinoma
B. Hematogenous dissemination
C. Lymphatic dissemination
D. Perineural spread
E. Surgical implantation

**Answers:**

A. Direct extension from primary colorectal carcinoma – Incorrect. The cutaneous metastasis recurred from a previously excised and treated primary colorectal adenocarcinoma, making direct extension unlikely. Additionally, the tumor is visualized within the lymphatic spaces on histology.
B. Hematogenous dissemination – Incorrect. While this is a plausible theory for cutaneous metastasis, this is unlikely given the presence of tumor visualized within the lymphatic spaces on histology.^5^
C. Lymphatic dissemination – Correct. The presence of tumor present within the lymphatic spaces on histology is strongly supportive of this mode of dissemination. The distribution of the lymphangioma-like vesicles and ecchymotic plaques follows the distribution of the superficial inguinal lymph nodes, explaining the metastatic pattern from the previous colorectal adenocarcinoma.
D. Perineural spread – Incorrect. Perineural spread has been previously postulated as a mechanism of zosteriform patterned cutaneous metastasis. However, this is unlikely given tumor visualized within the lymphatic spaces on histology.^5^
E. Surgical implantation – Incorrect. The bilateral inguinal regions were not involved in the abdominoperineal resection, making this an unlikely reason for cutaneous metastasis.

## Conflicts of interest

None disclosed.
